# Prevalence of Influenza-Like Illness and Seasonal and Pandemic H1N1 Influenza Vaccination Coverage Among Workers — United States, 2009–10 Influenza Season

**Published:** 2014-03-14

**Authors:** Sara E. Luckhaupt, Geoffrey M. Calvert, Jia Li, Marie Sweeney, Tammy A. Santibanez

**Affiliations:** 1 Division of Surveillance, Hazard Evaluations, and Field Studies, National Institute for Occupational Safety and Health, CDC; 2 National Center for Immunization and Respiratory Diseases, CDC

During an influenza pandemic, information about the industry and occupation (I&O) of persons likely to be infected with influenza virus is important to guide key policy decisions regarding vaccine prioritization and exposure-control measures. Health-care personnel (HCP) might have increased opportunity for exposure to influenza infection, and they have been prioritized for influenza vaccination because of their own risk and the risk that infected HCP pose to patients (1). To identify other groups of workers that might be at increased risk for pandemic influenza infection, influenza-like illness (ILI) and vaccination coverage data from the 2009 National H1N1 Flu Survey (NHFS), which was conducted during October 2009 through June 2010, were analyzed. In a representative sample of 28,710 employed adults, 5.5% reported ILI symptoms in the month before the interview, and 23.7% received the 2009 pandemic H1N1 (pH1N1) influenza vaccine. Among employed adults, the highest prevalence of ILI was reported by those employed in the industry groups “Real estate and rental and leasing” (10.5%) and “Accommodation and food services” (10.2%), and in the occupation groups “Food preparation and serving related” (11.0%) and “Community and social services” (8.3%). Both seasonal influenza and pH1N1 vaccination coverage were relatively low in all of these groups of workers. Adults not in the labor force (i.e., homemakers, students, retired persons, and persons unable to work) had ILI prevalence and pH1N1 vaccination coverage similar to those found in all employed adults combined; in contrast, ILI prevalence was higher and pH1N1 vaccination coverage was lower among unemployed adults (i.e., those looking for work). These results suggest that adults employed in certain industries and occupations might have increased risk for influenza infection, and that the majority of these workers did not receive seasonal or pH1N1 influenza vaccine. Unemployed adults might also be considered a high risk group for influenza.

The NHFS was designed to produce population-based estimates of the prevalence of ILI and seasonal and pH1N1 influenza vaccination coverage during the 2009–10 influenza season, when the novel influenza A (H1N1) strain (influenza A [H1N1]pdm09 or pH1N1) was circulating at pandemic levels. As described elsewhere (2), the NHFS was a random-digit–dialed telephone survey that sampled landline telephone and cellular telephone households from all 50 states and the District of Columbia. In addition to questions related to influenza vaccination status and recent respiratory illness and health risks, the adult questionnaire included questions about employment status and I&O of employment. Monthly targets were set to achieve approximately 6,000 interviews per month. Interviews were conducted during October 2009 through June 2010. The Council of American Survey Research Organizations (CASRO) response rate* for the NHFS was 34.0% for landline telephone respondents and 25.5% for cellular telephone respondents.

ILI was defined as having been sick with fever and cough or sore throat in the past month. Adjusted prevalence and adjusted prevalence ratios (APRs) based on predicted marginals from logistic regression models are reported. Groups with relatively low prevalence of ILI and relatively high sample sizes were used as reference categories for APRs. Those who reported receiving the seasonal influenza vaccine during the period from August 2009 to the month of interview were defined as vaccinated against seasonal influenza, whereas those who reported receiving the 2009 pH1N1 vaccine during the period from October 2009 to the month of interview were considered vaccinated against 2009 pH1N1. Vaccination coverage estimates were calculated using the Kaplan-Meier survival analysis procedure to determine the cumulative proportion of persons vaccinated with at least 1 dose of each vaccine. For respondents who indicated they had been vaccinated but had a missing date of vaccination (5.8% for 2009 pH1N1 and 3.8% for seasonal influenza), the month and year of vaccination was imputed using the weighted sequential hot deck method. Results were weighted and analyzed with statistical software to account for the complex survey design. Influenza vaccination coverage estimates based on this survey for all adults and children have been published previously, in combination with data from the Behavioral Risk Factor Surveillance System (2).

Among employed adults, the highest prevalence of ILI was reported by those employed in the industry groups “Real estate and rental and leasing” (10.5% [95% confidence interval (CI) = 5.1%–20.5%]) and “Accommodation and food services” (10.2% [CI = 7.4%–13.9%]) (Table 1). In addition to these two groups, both the “Educational services” and “Manufacturing” industries had significantly higher APRs for ILI compared with the reference industry group of “Finance and insurance.” Among occupation groups, the highest prevalences of ILI were reported by “Food preparation and serving related” (11.0% [CI = 7.7%–15.5%]) and “Community and social services” (8.3% [CI = 4.2%–15.9%]) (Table 2). In addition to these two groups, “Personal care and service,” “Building and grounds cleaning and maintenance,” and four other groups had significantly higher APRs for ILI compared with the reference occupation group of “Business and financial operations.” In the “Accommodation and food services” industry and the “Food preparation and serving related” occupation group, coverage with both seasonal influenza vaccine and pH1N1 vaccine were lower than vaccination coverage among all employed adults combined (Table 3).

The APR for ILI for the industry group “Healthcare and social assistance” was not significantly different from 1.0, and neither were the APRs for ILI for the occupations of “Healthcare support” or “Healthcare practitioners and technical.” On the other hand, these industry and occupation groups reported the highest pH1N1 vaccination coverage (38.8%–58.7%) and, along with “Life, physical, and social science” occupations, the highest seasonal influenza vaccination coverage (47.2%–67.0%).

Among all adults, employed persons had a similar prevalence of ILI in the month before the interview (5.5%) compared with those not in the labor force (6.0%); these groups also had similar pH1N1 vaccination coverage (23.7% versus 26.5%) (Table 3). In contrast, ILI prevalence was higher (9.4%) and pH1N1 vaccination coverage was lower (16.7%) among unemployed adults in the labor force.

## Editorial Note

As part of a comprehensive influenza prevention program, the goals of worker vaccination and exposure control measures include 1) protecting the worker, and 2) protecting the public (e.g., patients, students, and customers). Health-care and emergency medical services personnel were one of the initial target groups for 2009 pH1N1 influenza vaccination (3). Although other specific groups of civilian workers have not been targeted for influenza vaccination based on industry or occupation, the Occupational Safety and Health Administration’s *Guidance for Preparing Workplaces for an Influenza Pandemic* (4) recognizes that occupational exposure to influenza during a pandemic “depends in part on whether or not jobs require close proximity to persons potentially infected with the pandemic influenza virus, or whether they are required to have either repeated or extended contact with known or suspected sources of pandemic influenza virus such as coworkers, the general public, outpatients, school children or other such individuals or groups.”

What is already known on this topic?

Workers are at risk for becoming infected with influenza from customers and coworkers in the workplace. During the early stages of an influenza pandemic, shortages of vaccine and personal protective equipment can occur, necessitating the prioritization of groups of workers for preventive interventions.

What is added by this report?

During the 2009–10 influenza season, when a global pandemic of novel influenza A (H1N1) was under way, both the prevalence of influenza-like illness in the prior month and the cumulative incidence of seasonal and 2009 pandemic H1N1 influenza (pH1N1) vaccination varied significantly by employment status and among workers in different industry and occupation groups. The highest prevalence of influenza-like illness symptoms was reported by those employed in the industry groups “Real estate and rental and leasing” (10.5%) and “Accommodation and food services” (10.1%), and in the occupation groups “Food preparation and serving related” (10.9%) and “Community and social services” (8.3%). These groups of workers had relatively low levels of both seasonal and pH1N1 influenza vaccination coverage.

What are the implications for public health practice?

Relatively high prevalence rates of influenza-like illness among workers who likely have high exposure to the public and among unemployed adults during the 2009–10 influenza season suggest that these groups might be at increased risk for infection during a pandemic. Employers should evaluate risk levels in workplace settings and implement control measures that include influenza vaccination programs, education on hand hygiene and cough etiquette, encouraging workers to stay home from work when ill, and provision of personal protective equipment.

This is one of the first reports to describe the prevalence of ILI among I&O groups other than HCP. The relatively high prevalence rates of ILI among workers employed in food service, education, community and social services, personal care, and cleaning and maintenance are consistent with the hypothesis that the risk for acquiring influenza in the workplace is highest for workers with frequent contact with the public and/or fomites and overlap with findings from previous studies (5,6). The high prevalence of ILI among workers in the “Real estate and rental and leasing” category was somewhat surprising; however, many of the workers in this industry are employed in “Sales and related” occupations, which might involve contact with infectious customers and fomites. The relatively low vaccination coverage among these I&O groups suggests that their potentially increased risk for infection is not being recognized by the workers themselves or by their employers, who could play a role in providing and promoting vaccination in the workplace.

The findings in this report are subject to at least four limitations. First, all results are based upon self-report, and neither illness nor vaccination status were validated with medical records; not all ILIs are influenza, and respondents might not have accurately reported which vaccine(s) they received. Second, survey bias might have resulted from the noninclusion of households with no telephone service and the low response rate; although weighting adjustments were made, some bias might remain. Third, differences in the prevalence of ILI and vaccination coverage among workers in different I&O categories might be confounded by other nonoccupational variables for which no adjustment was made (e.g., children in the home). Finally, broad I&O categories were used for this analysis. A drawback to using broad I&O categories is that they aggregate workers who likely have substantially different exposure levels.

Relatively high prevalence rates of ILI among workers who likely have high exposure to the public and among unemployed adults during the 2009–10 influenza season suggest that these groups might be at increased risk for infection during a pandemic. None of these non–health-care worker groups achieved high rates of seasonal or pH1N1 influenza vaccination coverage. On the other hand, the relatively high rates of vaccination coverage among HCP might have contributed to their relatively low rates of ILI. Employers should evaluate risk levels in workplace settings and implement prevention measures that include workplace influenza vaccination programs, education on hand hygiene and cough etiquette, encouraging workers to stay home from work when ill, and provision of personal protective equipment when appropriate. These measures will protect the workers and the public.

## Figures and Tables

**TABLE 1:** Influenza-like illness (ILI) and seasonal and 2009 pandemic influenza A (H1N1) (pH1N1) vaccination coverage, by industry of employment — 2009 National H1N1 Flu Survey, United States

Industry category (2007 NAICS code)	Unweighted sample size	Weighted prevalence of ILI		Adjusted PR for ILI		Seasonal influenza vaccination coverage		pH1N1 influenza vaccination coverage	
			
		%	(95% CI)*	PR	(95% CI)†§	%	(95% CI)¶	%	(95% CI)**
Real estate and rental and leasing (NAICS 53)	449	10.5	(5.1–20.5)	3.31	(1.49–7.38)	35.9	(27.4–44.4)	16.8	(10.7–22.9)
Accommodation and food services (NAICS 72)	1,128	10.2	(7.4–13.9)	3.12	(1.91–5.11)	17.4	(12.6–22.2)	16.5	(12.1–20.9)
Educational services (NAICS 61)	3,800	6.3	(5.0–8.0)	1.91	(1.23–2.96)	43.6	(40.5–46.7)	26.8	(23.9–29.7)
Information (NAICS 51)	663	6.1	(3.2–11.1)	1.89	(0.92–3.88)	30.3	(24.2–36.4)	11.8	(7.8–15.8)
Manufacturing (NAICS 31–33)	1,844	5.6	(4.0–8.0)	1.77	(1.08–2.91)	37.6	(33.5–41.7)	18.6	(14.5–22.7)
Administrative and support and waste management and remediation services (NAICS 56)	704	5.5	(3.0–10.0)	1.65	(0.79–3.41)	22.5	(17.2–27.8)	13.2	(9.0–17.4)
Health care and social assistance (NAICS 62)	5,185	5.4	(4.4–6.7)	1.49	(0.96–2.30)	58.5	(55.8–61.2)	44.5	(41.1–47.9)
Retail trade (NAICS 44–45)	2,235	5.1	(3.9–6.7)	1.51	(0.96–2.39)	31.8	(28.2–35.4)	16.0	(12.8–19.2)
Public administration (NAICS 92)	1,870	5.1	(3.7–6.9)	1.39	(0.85–2.26)	48.0	(43.3–52.7)	29.3	(24.6–34.0)
Arts, entertainment, and recreation (NAICS 71)	585	5.1	(2.6–9.8)	1.55	(0.72–3.31)	32.8	(22.1–43.5)	16.9	(11.0–22.8)
Other services (except public administration) (NAICS 81)	1,163	5.0	(3.3–7.6)	1.63	(0.94–2.83)	24.8	(20.5–29.1)	14.4	(10.6–18.2)
Construction (NAICS 23)	1,822	4.9	(3.5–6.8)	1.49	(0.91–2.44)	21.6	(17.7–25.5)	11.8	(8.7–14.9)
Transportation and warehousing (NAICS 48–49)	1,081	4.5	(2.9–6.9)	1.41	(0.80–2.48)	25.6	(21.5–29.7)	13.7	(10.1–17.3)
Professional, scientific, and technical services (NAICS 54)	2,586	4.1	(3.2–5.3)	1.26	(0.81–1.98)	36.9	(33.5–40.3)	20.7	(17.5–23.9)
Utilities (NAICS 22)	270	4.0	(1.9–8.2)	1.31	(0.58–2.94)	36.6	(27.0–46.2)	16.0	(9.1–22.9)
Finance and insurance (NAICS 52)	1,330	3.8	(2.5–5.5)	Referent	—	39.7	(34.6–44.8)	15.2	(11.5–18.9)
Wholesale trade (NAICS 42)	440	3.8	(1.8–7.9)	1.17	(0.52–2.60)	34.0	(24.6–43.4)	21.4	(10.9–31.9)
Agriculture, forestry, fishing, and hunting (NAICS 11)	622	3.2	(1.6–6.3)	0.91	(0.42–2.00)	25.2	(18.1–32.3)	13.1	(8.0–18.2)
Mining, quarrying, and oil and gas extraction (NAICS 21)	177	2.3	(0.9–5.7)	0.79	(0.30–2.09)	25.2	(11.4–39.0)	23.7	(5.3–42.1)

**Abbreviations:** NAICS = North American Industry Classification System; PR = prevalence ratio; CI = confidence interval.

* Adjusted for interview month.

† Adjusted for interview month, vaccination status (seasonal and pH1N1 influenza), chronic medical conditions (asthma or another lung condition, diabetes, a heart condition, a kidney condition, sickle cell anemia or other anemia, a neurologic or neuromuscular condition, a liver condition, or a weakened immune system caused by a chronic illness or by medicines taken for a chronic illness), and age group.

§ Reference group is “finance and insurance” (NAICS 52).

¶ September 2009–June 2010.

** October 2009–June 2010.

**TABLE 2:** Influenza-like illness (ILI) and seasonal and 2009 pandemic influenza A (H1N1) (pH1N1) vaccination coverage, by occupation — 2009 National H1N1 Flu Survey, United States

Occupational group (2010 SOC major group)	Unweighted sample size	Weighted prevalence of ILI		Adjusted PR for ILI		Seasonal influenza vaccination coverage		pH1N1 influenza vaccination coverage	
			
		%	(95% CI)*	PR	(95% CI)†§	%	(95% CI)¶	%	(95% CI)**
Food preparation and serving related occupations (SOC 35)	836	11.0	(7.7–15.5)	3.07	(1.85–5.08)	21.2	(15.6–26.8)	15.6	(11.4–19.8)
Community and social services occupations (SOC 21)	695	8.3	(4.2–15.9)	2.26	(1.06–4.83)	45.0	(37.3–52.7)	30.4	(23.2–37.6)
Personal care and service occupations (SOC 39)	721	7.5	(4.5–12.3)	2.06	(1.11–3.83)	33.8	(26.7–40.9)	17.4	(12.6–22.2)
Building and grounds cleaning and maintenance occupations (SOC 37)	722	7.4	(4.1–12.9)	2.04	(1.04–4.02)	29.6	(23.8–35.4)	20.7	(13.9–27.5)
Healthcare support occupations (SOC 31)	631	7.0	(4.5–10.8)	1.36	(0.76–2.43)	47.2	(39.6–54.8)	38.8	(30.6–47.0)
Computer and mathematical occupations (SOC 15)	964	6.8	(4.5–10.3)	1.93	(1.12–3.30)	36.0	(30.7–41.3)	20.5	(15.9–25.1)
Production occupations (SOC 51)	1,019	6.6	(4.3–9.8)	1.95	(1.14–3.31)	31.9	(26.7–37.1)	15.6	(11.2–20.0)
Life, physical, and social science occupations (SOC 19)	486	6.3	(3.2–12.3)	1.66	(0.78–3.56)	52.8	(41.7–63.9)	34.8	(23.8–45.8)
Sales and related occupations (SOC 41)	2,344	6.2	(4.6–8.4)	1.69	(1.05–2.72)	29.9	(26.6–33.2)	16.3	(13.3–19.3)
Management occupations (SOC 11)	3,695	5.9	(4.6–7.6)	1.68	(1.09–2.60)	37.5	(34.5–40.5)	22.7	(19.9–25.5)
Education, training, and library occupations (SOC 25)	2,696	5.6	(4.3–7.2)	1.54	(0.99–2.40)	43.0	(39.4–46.6)	25.8	(22.7–28.9)
Construction and extraction occupations (SOC 47)	1,119	5.2	(3.3–8.0)	1.41	(0.80–2.48)	20.4	(15.6–25.2)	11.7	(7.5–15.9)
Legal occupations (SOC 23)	492	4.7	(2.6–8.4)	1.29	(0.64–2.62)	41.2	(32.4–50.0)	23.4	(15.1–31.7)
Office and administrative support occupations (SOC 43)	3,240	4.4	(3.5–5.5)	1.13	(0.74–1.72)	37.3	(33.7–40.9)	21.8	(16.6–27.0)
Transportation and material moving occupations (SOC 53)	1,006	4.3	(2.9–6.4)	1.26	(0.73–2.15)	23.0	(18.3–27.7)	13.4	(9.6–17.2)
Installation, maintenance, and repair occupations (SOC 49)	612	4.3	(2.6–7.0)	1.27	(0.69–2.32)	36.3	(28.8–43.8)	17.0	(10.4–23.6)
Healthcare practitioners and technical occupations (SOC 29)	2,591	3.9	(2.9–5.2)	1.00	(0.62–1.60)	67.0	(63.3–70.7)	50.7	(46.6–54.8)
Architecture and engineering occupations (SOC 17)	752	3.8	(1.8–7.6)	1.08	(0.48–2.40)	32.7	(27.4–38.0)	17.6	(13.1–22.1)
Business and financial operations occupations (SOC 13)	1,588	3.7	(2.6–5.2)	Referent	—	37.1	(32.8–41.4)	17.9	(13.8–22.0)
Farming, fishing, and forestry occupations (SOC 45)	225	3.1	(1.1–8.4)	0.57	(0.22–1.49)	28.0	(15.5–40.5)	14.3	(5.5–23.1)
Protective service occupations (SOC 33)	494	2.8	(1.3–5.8)	0.55	(0.25–1.24)	45.5	(34.8–56.2)	35.5	(26.4–44.6)
Arts, design, entertainment, sports, and media occupations (SOC 27)	737	2.0	(1.1–3.7)	0.57	(0.27–1.17)	31.4	(25.1–37.7)	14.8	(9.9–19.7)

**Abbreviations:** SOC = Standard Occupational Classification; PR = prevalence ratio; CI = confidence interval.

* Adjusted for interview month.

† Adjusted for interview month, vaccination status (seasonal and pH1N1 influenza), chronic medical conditions (asthma or another lung condition, diabetes, a heart condition, a kidney condition, sickle cell anemia or other anemia, a neurologic or neuromuscular condition, a liver condition, or a weakened immune system caused by a chronic illness or by medicines taken for a chronic illness), and age group.

§ Reference group is “business and financial operations occupations” (SOC 13).

¶ September 2009–June 2010.

** October 2009–June 2010.

**TABLE 3:** Influenza-like illness (ILI) and seasonal and 2009 pandemic influenza A (H1N1) (pH1N1) vaccination coverage, by employment status — 2009 National H1N1 Flu Survey, United States

Employment status	Unweighted sample size	Weighted prevalence of ILI		Adjusted PR for ILI		Seasonal influenza vaccination coverage		pH1N1 influenza vaccination coverage	
			
		%	(95% CI)*	PR	(95% CI)†	%	(95% CI)§	%	(95% CI)¶
Employed	28,710	5.5	(5.0–6.0)	0.84	(0.72–0.99)	38.1	(37.0–39.2)	23.7	(22.6–24.8)
Unemployed	3,142	9.4	(7.1–12.3)	1.41	(1.04–1.92)	25.4	(22.2–28.6)	16.7	(13.7–19.7)
Not in labor force**	21,649	6.0	(5.3–6.6)	Referent	**—**	52.6	(51.1–54.1)	26.5	(25.1–27.9)
**Total (all adults)**	**56,656** ††	**5.9**	**(5.5–6.3)**	**—**	**—**	**41.8**	**(41.0–42.6)**	**23.9**	**(23.1–24.7)**

**Abbreviations:** PR = prevalence ratio; CI = confidence interval.

* Adjusted for interview month.

† Adjusted for interview month, vaccination status (seasonal and pH1N1 influenza), chronic medical conditions (asthma or another lung condition, diabetes, a heart condition, a kidney condition, sickle cell anemia or other anemia, a neurologic or neuromuscular condition, a liver condition, or a weakened immune system caused by a chronic illness or by medicines taken for a chronic illness), and age group.

§ September 2009–June 2010.

¶ October 2009–June 2010.

** Includes homemakers, students, retirees, and adults unable to work.

†† Includes 3,155 adults with missing values for employment status.

## Footnotes

* Information regarding the calculation of CASRO response rates available at http://c.ymcdn.com/sites/www.casro.org/resource/resmgr/docs/casro_on_definitions_of_resp.pdf.
